# Orchestrating Binding Interactions and the Emergence of Avidity Driven Therapeutics

**DOI:** 10.1021/acscentsci.3c00242

**Published:** 2023-03-16

**Authors:** Eden Kapcan, Benjamin P. M. Lake, Anthony F. Rullo

**Affiliations:** Department of Medicine, Department of Chemistry and Chemical Biology, McMaster University, Hamilton, Ontario L8S 4L8, Canada

**Figure 1:**
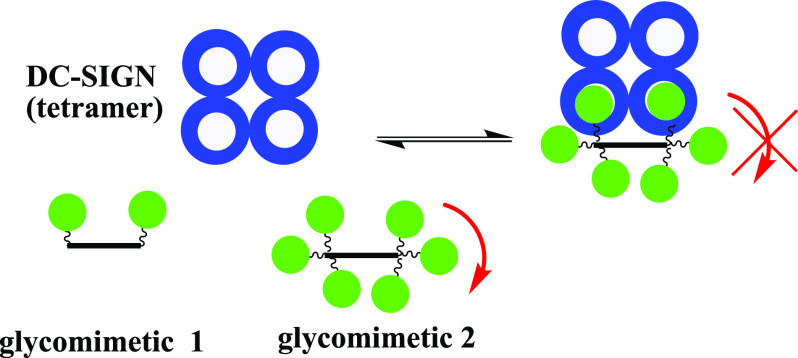
Avidity stabilized glycomimetic inhibitors for DC-SIGN studied in this work. Green spheres represent mannoside ligands; red arrows represent rotational degrees of freedom.

Avidity driven therapies aim to achieve exceptionally high target binding affinities by carefully integrating a number of weaker affinity binding ligands. This is in contrast to traditional medicinal chemistry strategies that aim to improve the binding affinity of a single ligand by optimizing its noncovalent (or covalent) interaction with a binding cleft on the target protein of interest. In addition to achieving high target binding affinity, these therapies can benefit from impressively high selectivity for the target of interest, whose copy number on a cell (e.g., tumor antigen expression) or number of binding sites (e.g., tetrameric lectin) complements the binding ligand arrangement on the avidity therapeutic. To successfully develop such a therapeutic, several lower affinity binding ligands must be carefully arranged spatially, to maximize the probability of making one or several contacts with the target. In this issue of *ACS Central Science*, Fieschi, Bernardi, and co-workers^1^ reveal key design principles and validation strategies to guide the development of high avidity binding therapies. Importantly, their results provide a general framework to design therapeutics that use minimal binding ligands (i.e., minimal multivalency) to achieve high affinity binding to target lectins. In this work, the authors perform systematic and rigorous thermodynamic analysis of dendrimeric glycomimetic ligand interactions with the tetrameric C-type lectin receptor DC-SIGN. DC-SIGN is a receptor implicated in a number of biological recognition processes, including SARS-COV-2 viral entry. Common to the general design of multivalent probes and inhibitors, these glycomimetic ligands present an array of multiple low affinity (synthetic mannoside) binding ligands to engage the target biomolecule (e.g., DC-SIGN), with high avidity. The fundamental goal of this study was to understand how and why relatively subtle changes in the spacing and valency of mannoside ligands on the glycomimetic can significantly affect its avidity for DC-SIGN. Understanding the molecular origins of binding avidity is highly complex given the interplay of multiple contributing factors, namely, chelation binding, receptor clustering, and statistical rebinding events/multiple binding modes (Figure 1). Collectively, these factors increase the probability that a binding ligand exists in a “bound state” with the target protein/cell surface relative to a free “unbound state”.

In this work, the authors perform structure/function analysis on a series of multivalent glycomimetic scaffolds that differ in the number (i.e., valency) and spacing of mannoside binding ligands. Their experimental design enabled assessment of the relative contributions of chelation binding, statistical rebinding events, and receptor (DC-SIGN) flexibility on binding avidity. It was observed that the greatest gains in binding affinity were achieved when (1) the glycomimetic sufficiently preorganized binding ligands for chelation binding and (2) binding ligands were arranged to increase statistical rebinding events (Figure 1, glycomimetic 2). Chelation binding occurs when two binding ligands are optimally spaced and can simultaneously engage two binding sites on the DC-SIGN tetramer; i.e., binding of the first ligand partially or fully pays the entropic cost for binding of the second ligand (Figure 1, both glycomimetics 1 and 2). In addition to chelation binding, each ligand within a cluster can “take turns” occupying a given single binding site, which increases the number of potential ligand rebinding events and binding modes (Figure 1, glycomimetic 2). Additionally, DC-SIGN flexibility was predicted by molecular modeling to increase the number of potential binding modes (i.e., the number of ways the glycomimetic can engage two binding sites). The significant contributions of statistical rebinding events to the overall strength of binding avidity validate previous reports by D. R. Bundle et al., describing this molecular phenomenon as a gain in “avidity entropy”.^2^ Here, the concept of avidity entropy was used to account for the observed high (sub-nanomolar) binding affinity between a multivalent carbohydrate inhibitor and pentameric protein toxin, whose interaction is favored by the potential for multiple statistical rebinding events.

In the current study,^1^ thermodynamic characterization of the highest affinity glycomimetic **2** by ITC revealed significant enthalpic stabilization that was partially compensated by entropic destabilization, when compared to its analogue **1** (Figure 1). Notably, both glycomimetics are preorganized for chelation binding; however, **2** has increased ligand valency (Figure 1). The entropic destabilization of **2** was partially attributed to a greater loss in the degrees of freedom upon binding DC-SIGN (since only one ligand can bind at a time, with the remaining two ligands of the rotor becoming frozen).

The current study^1^ synergizes with emerging efforts in translational chemical biology to design avidity binding probes and molecular therapeutics. One major current focus is to target single oligomeric receptors with multiple binding sites to block pathogenic infection. Such therapeutic modalities are especially useful when the goal is to inhibit an intrinsically high avidity interaction itself (e.g., influenza adhesion via hemagglutinin proteins to sialic acid on host cells). A recent key example targets HIV-1 envelope spike proteins using bivalent DNA-Fab conjugates that can simultaneously bind two of three binding sites on the spike trimer protein and block HIV-1 infection.^3,4^ Notably, the results of these studies also reveal the importance of a rigid linker to preorganize the binding ligands for efficient chelation binding. In this work, the binding ligands are strategically tethered to this rigid linker through a short flexible strand, analogous to the short PEG-like chains connecting mannoside binding ligands in the current glycomimetic study. This feature provides the necessary trade off in enthalpy/entropy compensation since it is very difficult to preorganize the two binding ligands perfectly for chelation binding. Too short or long a rigid linker will drastically destabilize bivalent binding enthalpically. Too flexible a linker, however, can incur significant entropic cost depending on the number of rotatable bonds in the linker that become frozen post binding. The findings of the current study^1^ also suggest the HIV-1 targeting conjugate and other avidity targeting conjugates for viral spike proteins may benefit from additional degenerate binding ligands and alternative topologies to increase statistical rebinding events.

**Figure 2:**
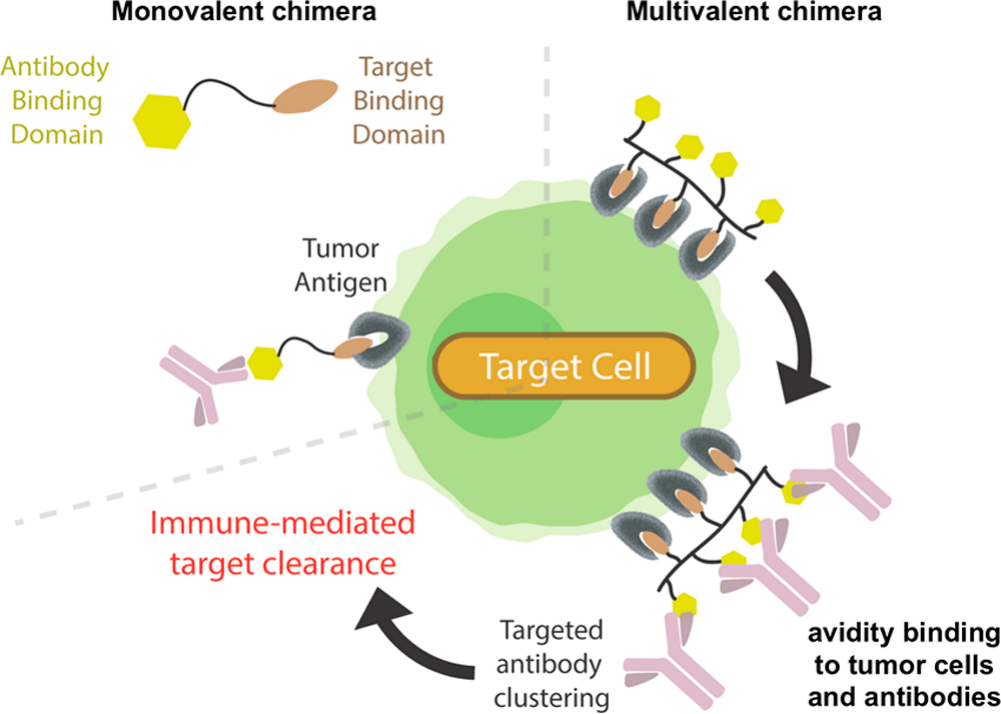
Multivalent chimera strategy using avidity to enhance immune recognition of tumor cells. Reproduced with permission from ref (8). Copyright 2023 John Wiley & Sons.

A second major current focus in the development of avidity binding probes and molecular therapeutics aims to target multiple receptors on a cell surface simultaneously. This is often done with the goal of selectively clustering and activating biological receptors to affect a specific cellular response,^5,6^ or targeting a diseased cell that overexpresses a key protein of interest.^7,8^ Lake et al. reported the development of multivalent tumor targeting chimeras that efficiently recruit serum antibodies to the surface of tumor cells, with the goal of inducing antitumor immune responses.^8^ These chimeras consisted of multiple tumor antigen and serum antibody binding ligands decorating a polymer backbone, and were observed to bind tumor cells with significantly longer residence times, compared to monovalent chimera analogues (Figure 2). These findings were consistent with rapid ligand rebinding events between tumor binding ligands on the multivalent chimera and an array of surface antigens on the tumor cell surface, not possible with monovalent analogues. Interestingly, monovalent chimeras precomplexed to IgG antibodies can bind cell lines with high avidity when cells are engineered to artificially express high levels of surface antigen. One chimera bound to each Fab of IgG generates a bivalent binding chimera:antibody complex. On normal cancer cell lines, however, when surface antigen expression levels are lower and antigens are likely farther apart, this avidity enhancement was lost. This was in contrast to what was observed with multivalent chimeras which can presumably contact surface antigens spaced farther apart and maintain avidity binding.

Considering the potential impact of DC-SIGN flexibility on glycomimetic avidity, it would be interesting to consider the role of membrane fluidity and the ability of surface antigens to transverse the plasma membrane in two dimensions on binding avidity in these cell targeting applications.

The development of avidity targeting therapeutics is likely to accelerate in future drug discovery programs at both academic and industrial levels. High avidity binding inhibitors can in principle be efficiently developed using highly accessible lower affinity binding ligands like the simple disaccharides used in the current study.^1^ This can potentially avoid the extensive medicinal chemistry required to generate highly potent small molecule binding ligands. In practice, however, this is highly nontrivial and requires careful design and biophysical experimentation as illustrated in the current body of work.^1^ As described above, multivalent ligands can benefit from multiple binding modes; however, this same binding event necessarily orders linker regions connecting the ligands, introducing conformational entropic cost. Future efforts to overcome entropic cost might benefit from creative solutions to couple high avidity therapeutic binding to the simultaneous “un-freezing” of constrained molecular elements. Inspiration for these designs might be found in nature, where proteins are known to interact with concomitant conformation changes in distal domains toward a more flexible disordered state, helping to offset the entropic cost of protein–protein binding.^9^
